# Submandibulectomy for Lithiasic Submandibular Sialadenitis: A Case Series of 23 Patients in the Drâa-Tafilalet Region of Southeastern Morocco

**DOI:** 10.7759/cureus.112555

**Published:** 2026-07-12

**Authors:** Abdelouahid Taleuan, Mohammed ElAmine Squalli Houssaini, Adnane Lakhal, Abdellatif Oudidi

**Affiliations:** 1 Otolaryngology - Head and Neck Surgery, Faculty of Medicine and Pharmacy of Errachidia, Al Amir Sultan Hospital, Errachidia, MAR; 2 Otolaryngology - Head and Neck Surgery, Centre Hospitalier Universitaire Hassan II, Fez, MAR

**Keywords:** drâa-tafilalet, lithiasic, salivary gland malignancy, sialolithiasis, submandibular gland, submandibulectomy

## Abstract

Background and objective: Submandibular sialolithiasis is the most frequent cause of salivary gland obstruction. Although gland-preserving procedures are increasingly used, submandibulectomy remains indicated in advanced, intra-glandular, recurrent, or complicated lithiasic sialadenitis. It is a safe, effective, and definitive therapeutic option in patients with advanced lithiasic submandibular sialadenitis. In resource-limited, geographically remote settings such as the Drâa-Tafilalet region of southeastern Morocco, patients often present at an advanced stage due to limited access to specialized care, rendering conservative or endoscopic approaches unfeasible. This study describes our surgical experience and underscores the necessity of early diagnosis and routine histopathological examination. The primary objective of this study was to describe the clinical profile, surgical management, post-operative outcomes, and histopathological findings of consecutive patients undergoing submandibulectomy for lithiasic submandibular sialadenitis in the Drâa-Tafilalet region. Secondary objectives were to describe the regional healthcare factors associated with delayed presentation and to emphasize the value of routine histopathological examination of all excised glands. Given the descriptive design and limited sample size, the study was not intended to determine the incidence of occult malignancy or establish causal relationships between regional factors and disease occurrence.

Methods: We conducted a retrospective descriptive study over a two-year period (January 2024-December 2025) in the Department of Otolaryngology - Head and Neck Surgery at Al Amir Sultan Hospital, Errachidia, Morocco. A total of 23 patients who underwent submandibulectomy for lithiasic submandibular sialadenitis were included. Epidemiological, clinical, radiological, therapeutic, and histopathological data were collected and analyzed.

Results: All 23 patients (male-to-female sex ratio of 13:10; mean age 51.1 years) underwent submandibulectomy. Mean symptom duration was 24.2 months. Histopathological examination revealed chronic sialadenitis in 21 (91.3%) cases and unexpected malignancy in two (8.7%) cases as follows: one low-grade mucoepidermoid carcinoma and one adenoid cystic carcinoma.

Conclusion: In Drâa-Tafilalet, lithiasic submandibular sialadenitis may reach late stages before specialist management. Submandibulectomy provided definitive disease control with acceptable morbidity in our series of 23 patients. Despite advances in gland-preserving approaches, it remains an important therapeutic option for advanced lithiasic disease in low-resource settings.

## Introduction

Salivary gland lithiasis (sialolithiasis) is the most common cause of obstructive salivary gland disease, accounting for approximately 50% of all major salivary gland pathology, with an estimated prevalence of 1.2% in the general population. The submandibular gland is affected in 80-90% of cases [1,2].

Current management of submandibular sialolithiasis increasingly relies on minimally invasive, gland-preserving techniques. Sialendoscopy, extracorporeal lithotripsy, transoral duct surgery, and combined approaches have markedly reduced the need for gland resection, avoiding the morbidity associated with open surgery. However, these treatment modalities are most effective when patients present early and require specialized equipment and experienced operators, limiting their widespread availability [2,3].

The Drâa-Tafilalet region in southeastern Morocco is a predominantly rural area characterized by limited access to specialized healthcare and environmental conditions that may promote dehydration and reduce salivary flow. These factors may contribute to delayed presentation, and patients frequently reach our provincial hospital with chronic submandibular sialadenitis, recurrent suppuration, or irreversible glandular fibrosis, often making submandibulectomy the only viable treatment option. Another important diagnostic challenge is the coexistence of salivary gland neoplasms with sialolithiasis. The radiological identification of a sialolith may mask an underlying neoplasm, which often remains undetected until histopathological examination [4,5].

## Materials and methods

Study design and setting

This retrospective descriptive study included all patients who underwent submandibulectomy for lithiasic submandibular sialadenitis at the Department of Otolaryngology - Head and Neck Surgery of Al Amir Sultan Hospital in Errachidia between January 1, 2024, and December 31, 2025.

Inclusion and exclusion criteria

All patients with a clinical, biological, and radiological diagnosis of submandibular sialolithiasis who underwent submandibulectomy during the study period, regardless of age or sex, were included. Patients with isolated anterior duct stones treated by simple transoral extraction without gland excision, non-lithiasic autoimmune sialadenitis, primary neoplasms diagnosed pre-operatively, radiation-induced sialadenitis, or incomplete medical records were excluded.

Pre-operative assessment

All patients underwent a standardized pre-operative workup including detailed clinical history (symptom duration, number of infectious episodes, prior antibiotic treatments), clinical examination (submandibular swelling, bimanual palpation of Wharton's duct, floor-of-mouth examination, assessment of the oral mucosa and dentition, and cervical lymphadenopathy), routine blood tests, and radiological assessment. Imaging consisted of panoramic dental radiograph and cervical ultrasound in all cases; computed tomography (CT) was performed in cases with suspected deep extension, trismus, or atypical features. Magnetic resonance imaging (MRI) was not performed in any of our patients, and sialendoscopy was not available at our center.

Surgical technique

All procedures were performed under general anesthesia by the same surgical team. A standard cervical approach was employed as follows: horizontal incision two finger-breadths below the inferior border of the mandible, skin flap elevation, identification and preservation of the marginal mandibular branch of the facial nerve, ligation of the facial artery and vein, dissection and delivery of the submandibular gland, with attention to the lingual and hypoglossal nerves, and ligation and transection of Wharton's duct as proximally as possible.

Histopathological examination

All excised glands were submitted for systematic histopathological examination, regardless of the pre-operative clinical or radiological impression. Specimens were fixed in 10% formalin, processed routinely, and examined by a senior pathologist using standard hematoxylin and eosin staining; additional sections or immunohistochemical staining were performed when histological features raised suspicion of an underlying neoplastic process.

Post-operative follow-up and data collection

Patients were reviewed at one week, one month, three months, and six months post-operatively. Data collected included operative duration, intra-operative complications, post-operative complications, histopathological results, need for adjuvant treatment, and oncological follow-up where applicable. Statistical analysis was descriptive. Patients were identified through the departmental operative registry and corresponding medical records. Clinical, radiological, operative, and follow-up data were extracted retrospectively from standardized medical charts. Regional variables, including travel distance, referral pathway, and hydration habits, were collected from information documented during routine clinical assessment. No significant missing data affected the variables included in the descriptive analysis.

Statistical analysis

Data were analyzed using descriptive statistics. Categorical variables were expressed as frequencies and percentages, and continuous variables were expressed as means with standard deviations and ranges. All statistical analyses were performed using Microsoft Excel 2019 (Redmond, WA: Microsoft Corp.).

Ethical considerations

This study was conducted in accordance with institutional ethical standards. Patient confidentiality was strictly maintained, and no personal identifiers were disclosed in the analysis. As this was a retrospective observational study using anonymized data, formal ethics committee approval was not required according to our institutional policy.

Regional context: particularities of the Drâa-Tafilalet area

Several sociogeographical factors specific to this region were systematically recorded as follows: distance from the patient's residence to the hospital, number of previous consultations at primary care level, reasons for delayed referral, and dietary habits. Dehydration secondary to extreme heat exposure (summer temperatures regularly exceeding 45°C), heavy consumption of high-mineral-content well water, and low daily water intake were identified as potential regional lithogenic factors.

## Results

Epidemiological data

A total of 23 patients met the inclusion criteria. The cohort comprised 13 (56.5%) men and 10 (43.5%) women, yielding a male-to-female ratio of 1.3:1. The mean age was 51.1±9.8 years (range: 35-71 years). A total of 11 (47.8%) patients were over 55 years of age. All patients resided in the Drâa-Tafilalet region; 18 (78.3%) lived in rural localities more than 80 km from the hospital. The mean distance from home to the referral center was 142±67 km.

Clinical presentation

The mean duration of symptoms before consultation at our department was 24.2±8 months (range: five to 48 months). All patients had been seen previously at the primary care level; 17 (73.9%) had received at least two courses of antibiotics for recurrent infectious episodes before referral. The predominant symptoms were as follows: recurrent painful submandibular swelling exacerbated by meals (22 patients, 95.7%), purulent episodes or frank abscess requiring drainage (14 patients, 60.9%), trismus (two patients, 8.7%), and progressive induration of the gland with or without associated floor-of-mouth involvement (18 patients, 78.3%). No patient was asymptomatic at presentation.

Notably, two patients (both elderly males aged 63 and 71 years, respectively) presented with a particularly indurated, non-tender submandibular mass associated with prolonged constitutional symptoms that had initially been attributed to chronic infection. In both patients, imaging confirmed sialolithiasis, and the clinical presentation was therefore primarily attributed to lithiasic sialadenitis. Histopathological examination subsequently revealed an associated malignant salivary gland tumor.

Imaging findings

Cervical ultrasound was performed in all 23 patients and confirmed the presence of submandibular gland enlargement with intraparenchymal or ductal hyperechoic calcifications in all cases. Stone size ranged from 5 to 19 mm (mean: 8.9±2.7 mm). CT scan of the neck was performed in eight (34.8%) patients, predominantly those with suspected deep extension, trismus, or atypical clinical features, and confirmed the ultrasound findings while providing better delineation of the extent of the inflammatory process. No pre-operative MRI was performed. Fine-needle aspiration cytology (FNAC) was not carried out in any patient.

Surgical treatment

**Figure 1:**
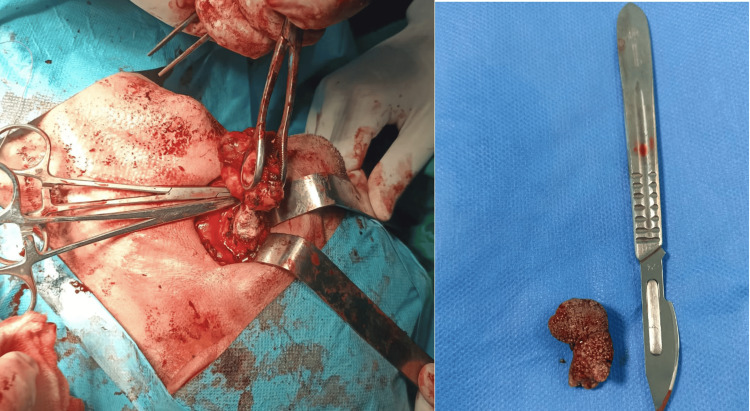
Surgical specimen showing the excised submandibular gland and a giant sialolith.

**Figure 2:**
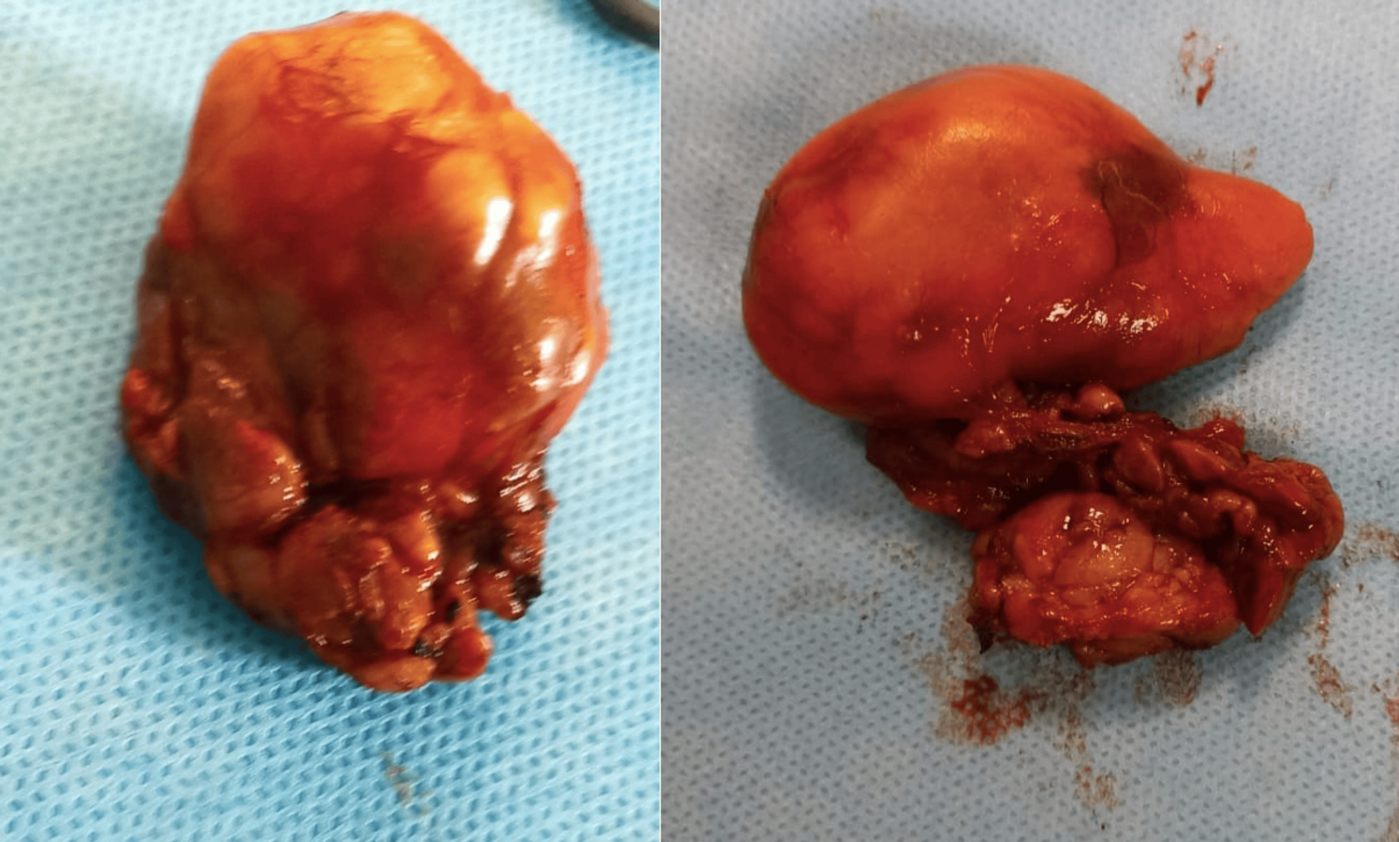
Macroscopic appearance of the resected submandibular glands, both showing features suspicious for malignancy.

**Figure 3:**
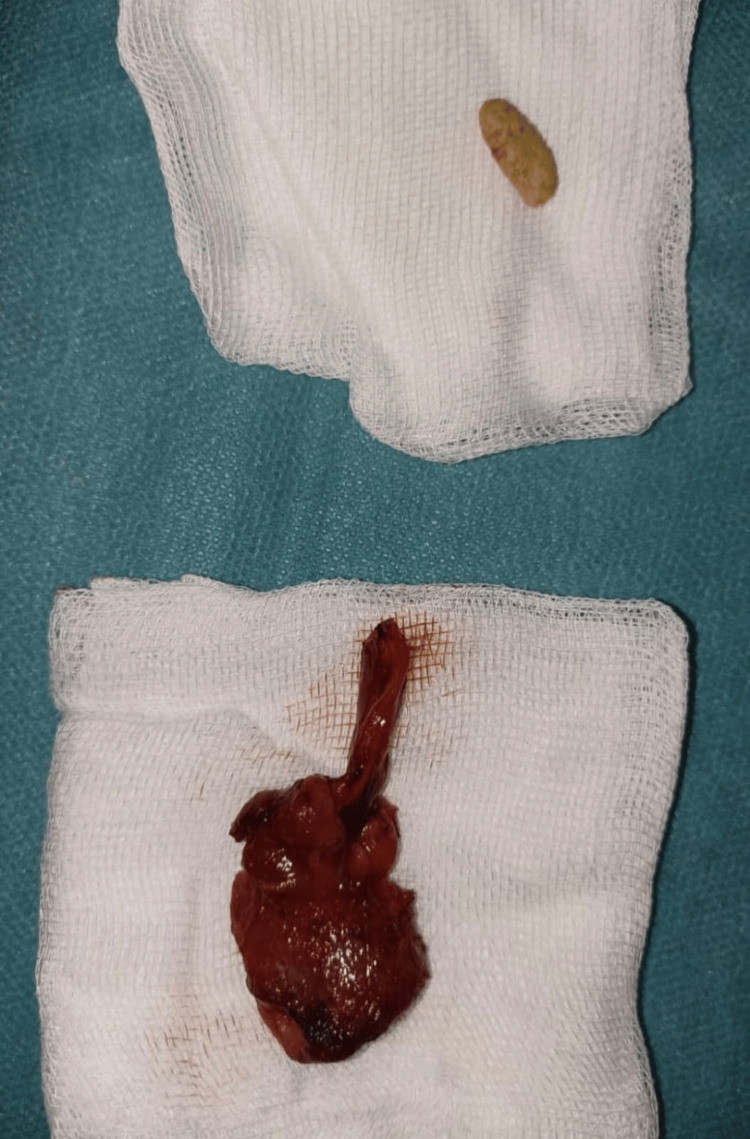
Macroscopic appearance of the extracted sialolith and the submandibular gland after surgical removal.

All 23 patients underwent total submandibulectomy - 13 (56.5%) on the right side and 10 (43.5%) on the left. Mean operative time was 44±12.1 min. No intra-operative complications were recorded. A closed-suction drain was placed in all patients and removed at 24 h post-operatively (Figures 1-3).

Post-operative outcomes and complications

Post-operative complications occurred in two (8.7%) patients. One (4.3%) patient developed transient marginal mandibular nerve paresis, which resolved spontaneously within three months without residual deficit. One (4.3%) patient presented with superficial wound infection successfully treated by local wound care and oral antibiotics. No hematoma, salivary fistula, or lingual nerve injury was recorded. Mean hospital stay was 2.2 days. All patients were satisfied with their post-operative evolution at one-month follow-up.

Histopathological findings

Systematic anatomopathological examination of the surgical specimen was performed in all 23 cases. The results were as follows: in 21 (91.3%) patients, histology confirmed chronic sclerosing sialadenitis with fibrosis or parenchymal atrophy secondary to ductal obstruction by the calculus - concordant with the pre-operative diagnosis. In four of these, active acute-on-chronic inflammation with microabscess formation was also noted. In two (8.7%) patients, histological examination revealed an unsuspected associated malignancy as follows: low-grade mucoepidermoid carcinoma of the submandibular gland, pT2N0M0 (TNM eighth edition). The tumor measured 2.2 cm in greatest dimension and was completely excised with clear margins. The patient was discussed in a multidisciplinary tumor board meeting; adjuvant radiotherapy was recommended. Adenoid cystic carcinoma of the submandibular gland, pT2N0M0. Tumor size was 2.1 cm. Complete surgical resection was achieved with negative margins. Adjuvant radiotherapy was delivered, given perineural invasion noted on histology. At last follow-up (12 months post-operatively), both patients were alive without clinical evidence of local recurrence.

## Discussion

The submandibular gland represents the predominant site of sialolithiasis, accounting for approximately 80-90% of reported cases. This anatomical predisposition can be attributed to the following three key pathophysiological mechanisms: the properties of submandibular saliva, characterized by its mucous, viscous composition due to elevated mucin content, which promotes calcium ion supersaturation and subsequent crystal nucleation within the ductal lumen. The anatomical trajectory of Wharton's duct, which follows an ascending course opposing gravitational forces, thereby impedes salivary drainage and predisposes to intra-ductal stasis. The morphological characteristics of the excretory duct itself, notably its narrow luminal diameter combined with its considerable length, collectively generate significant resistance to salivary outflow and facilitate progressive mineral deposition along the ductal walls [6,7]. To our knowledge, this series is among the first to report submandibulectomy outcomes specifically in the Drâa-Tafilalet region of Morocco. The demographic and clinical data we have gathered illuminate a pattern that diverges substantially from Western and urban Moroccan series.

The mean symptom duration of 24.2 months and the high rate of prior antibiotic treatments (73.9%) are striking indicators of diagnostic delay. This delayed consultation has similarly been reported by several African authors [8,9]. In contrast, reports from European tertiary referral centers describe earlier diagnosis and widespread use of minimally invasive techniques [10,11].

The region's hyper-arid climate, with summer temperatures frequently exceeding 45°C, may predispose the population to chronic dehydration and may contribute to stone formation by reducing salivary flow and increasing calcium and mucin concentration in ductal secretions [12,13]. The geographic barrier may also contribute to delayed referral, as nearly 80% of our patients lived more than 80 km from the hospital. Lithiasis-related submandibular sialadenitis may present in a destructive, chronic stage in Drâa-Tafilalet, leading directly to submandibulectomy rather than gland-preserving treatment.

The shift towards gland-preserving techniques in sialolithiasis management is undeniable. Sialendoscopy, first described by Marchal and Dulguerov and now widely adopted in specialized centers, achieves stone retrieval in 75-85% of cases while preserving glandular function; however, it requires specialized equipment, trained operators, and an adequate healthcare infrastructure [1]. Combined approaches associating transoral incision with endoscopic guidance further extend the indications. These techniques have considerably narrowed the indications for submandibulectomy in the management of sialolithiasis. Treatment selection is guided by a comprehensive assessment of the calculus characteristics, including its topographic distribution, dimensional parameters, and precise localization within the ductal system.

The extraoral approach to submandibular gland removal remains a viable option in the armamentarium for managing sialolithiasis, as it is a commonly used technique that has yielded satisfactory results, as shown in these cases. The major disadvantages are the less favorable esthetic results and the risk of transient or permanent paralysis of the marginal mandibular branch of the facial nerve [5]. The outcomes of submandibulectomy in our series, characterized by the absence of intra-operative complications and a transient nerve paresis rate of 4.3% with complete resolution within three months, substantiate the safety and efficacy profile of this procedure when performed by experienced surgical teams.

Similarly, in a retrospective study of 133 patients who underwent submandibular gland excision, Ichimura et al. reported that 87 cases presented with irreversible chronic inflammatory glandular disease [14]. Post-operative neurological complications following transcervical access, including marginal mandibular nerve palsy, lingual nerve paresthesia, and hypoglossal nerve dysfunction, were exclusively transient in nature. In our series, marginal mandibular nerve paresis resolved within two weeks, with no recorded cases of hypoglossal nerve palsy or lingual nerve paresthesia.

Salivary gland neoplasms can mimic inflammatory and obstructive salivary gland disease. Our rate of 8.7% is considerably higher than that reported in the literature, where occult malignancy in glands excised for presumed lithiasic submandibular sialadenitis is exceptionally rare. This observation should not be interpreted as reflecting the true prevalence of occult malignancy among patients with lithiasic submandibular sialadenitis, because of the limited sample size in our study. Most published series describe only chronic inflammatory changes, fibrosis, acinar atrophy, and ductal ectasia on histopathological analysis [15].

This study has several limitations. First, it is a retrospective single-center descriptive case series with a relatively small sample size, which limits the generalizability of our findings. Second, the absence of a comparison group precludes direct comparison with patients managed using gland-preserving techniques. Third, only patients who underwent submandibulectomy were included, which may introduce a potential selection bias toward more advanced disease. In addition, although delayed referral, dehydration, geographical isolation, and other regional factors may have contributed to advanced disease presentation, the retrospective design of this study does not allow causal relationships to be established. Finally, the unexpectedly high proportion of occult malignancies observed in our series should be interpreted with caution, given the limited number of patients, and should not be considered representative of the true prevalence in patients with lithiasic submandibular sialadenitis.

Our results make a compelling case for improved early detection of submandibular sialolithiasis in the Drâa-Tafilalet region. At the primary care level, clinicians should be trained to recognize the classic triad of recurrent pre-prandial submandibular swelling, palpable floor-of-mouth induration, and prior infectious episodes as a pattern requiring ENT referral. Simple tools, floor-of-mouth bimanual palpation, and a cervical ultrasound are sufficient to establish the diagnosis. Earlier referral would not only allow a proportion of patients to benefit from gland-preserving techniques at tertiary centers but also enable earlier detection of the small subgroup harboring underlying malignancy.

## Conclusions

This retrospective series of 23 submandibulectomies for lithiasic submandibular sialadenitis performed in the Drâa-Tafilalet region of southeastern Morocco illustrates the profound influence of geographic, socioeconomic, and healthcare access factors on the clinical presentation and management of a common salivary gland disease. The near-universal late presentation in our series rendered minimally invasive approaches inapplicable, making submandibulectomy an important therapeutic option for selected patients presenting with advanced disease in our setting. Also, the presence of sialolithiasis should not completely exclude an associated salivary gland malignancy.
